# Mental State Detection Using Riemannian Geometry on Electroencephalogram Brain Signals

**DOI:** 10.3389/fnhum.2021.746081

**Published:** 2021-11-26

**Authors:** Selina C. Wriessnegger, Philipp Raggam, Kyriaki Kostoglou, Gernot R. Müller-Putz

**Affiliations:** ^1^Institute of Neural Engineering, Graz University of Technology, Graz, Austria; ^2^BioTechMed-Graz, Graz, Austria; ^3^Research Group Neuroinformatics, Faculty of Computer Science, University of Vienna, Vienna, Austria; ^4^Department of Neurology and Stroke, Hertie Institute for Clinical Brain Research, University of Tübingen, Tübingen, Germany

**Keywords:** EEG, mental workload, mental fatigue, band power features, Riemannian geometry

## Abstract

The goal of this study was to implement a Riemannian geometry (RG)-based algorithm to detect high mental workload (MWL) and mental fatigue (MF) using task-induced electroencephalogram (EEG) signals. In order to elicit high MWL and MF, the participants performed a cognitively demanding task in the form of the letter *n*-back task. We analyzed the time-varying characteristics of the EEG band power (BP) features in the theta and alpha frequency band at different task conditions and cortical areas by employing a RG-based framework. MWL and MF were considered as too high, when the Riemannian distances of the task-run EEG reached or surpassed the threshold of the baseline EEG. The results of this study showed a BP increase in the theta and alpha frequency bands with increasing experiment duration, indicating elevated MWL and MF that impedes/hinders the task performance of the participants. High MWL and MF was detected in 8 out of 20 participants. The Riemannian distances also showed a steady increase toward the threshold with increasing experiment duration, with the most detections occurring toward the end of the experiment. To support our findings, subjective ratings (questionnaires concerning fatigue and workload levels) and behavioral measures (performance accuracies and response times) were also considered.

## Introduction

With increasing developments of modern technologies like artificial intelligence and virtual reality environments, more and more applications make use of mental state monitoring systems. These systems allow applications to be adapted based on the user’s mental state which is a crucial factor in fields such as driving or teaching assistance (Davidse et al., 2009; Walter et al., 2017; Zander et al., 2017).

Mental state changes, and more precisely increasing mental workload (MWL) and mental fatigue (MF), are known to affect the performance of a person while executing a cognitive demanding task (Käthner et al., 2014). This effect is usually projected on electrophysiological signals such as brain signals. MWL can be defined as the number of tasks to be performed simultaneously, the load in working memory, or more generally as a measure of the quantity of mental resources engaged in a task (Owen et al., 2005; Roy et al., 2013). Therefore, MWL can be seen as a measure of task difficulty and depends on each individual’s capabilities and effort (Gevins and Smith, 2006). High MWL may affect people who use technology in their everyday life, such as interacting with computers, smartphones, and other devices. Mental overload, as a result of high MWL, can compromise a user’s performance and even safety by increasing error rates and reaction times (Xie and Salvendy, 2000; Young and Stanton, 2002), and can lead to the neglection of critical information, known as cognitive tunneling (Thomas and Wickens, 2001; Dixon et al., 2013; Dehais et al., 2014). MF is both objective and subjective. In its subjective dimension, MF is described as the feeling of weariness and lack of energy due to prolonged periods of cognitive activity. From an objective point of view, it is associated with exhaustion or tiredness that leads to a decrease in task performance and commitment (Hockey, 1997; Boksem et al., 2005). High MF may also hinder the attempts of a user to complete a task that requires self-motivation, without signs, however, of cognitive failure or motor weakness (Chaudhuri and Behan, 2000). It has been shown that reduced motivation of a user to perform a task which induces high MF, is associated with increased sympathetic activity and decreased parasympathetic activity (Mezzacappa et al., 1998; Johnson et al., 2006; Tanaka et al., 2009).

The methods of detecting MWL and MF can be divided into three main categories: Self-reporting and subjective ratings, behavioral measures and (neuro)physiological measures (Aghajani et al., 2017). The subjective level of workload can be determined with the NASA Task Load Index (TLX) questionnaire (Hart and Staveland, 1988), whereas the subjective level of fatigue can be evaluated with the Visual Analog Scale to Evaluate Fatigue Severity (VAS-F) questionnaire (Lee et al., 1991). Studies of Käthner et al. (2014) and Roy et al. (2013) have shown that if there is an increase in MWL and MF, there will be an increase of the subjective levels of workload and fatigue in the participants. Another behavioral method is to measure primary- and secondary-task performance, such as performance accuracy and reaction time (Wickens, 2008). If there is an increase in MWL and MF, there will be a decrease in the task performance (decrease of the accuracy and an increase of the reaction time) of the participants (Roy et al., 2013; Käthner et al., 2014). Accuracy and reaction time of the participants can be recorded during the experiment and evaluated afterward. And finally, the (neuro)physiological measures of MWL and MF are heart rate variability, oculomotor activity (eye movements), pupillometry (measure of pupil size and reflexes), electromyography (electrical activity produced by skeletal muscles), galvanic skin responses (changes in sweat gland activity), and brain activity (Sahayadhas et al., 2012). The (neuro)physiological measure for MWL and MF detection used in this study is brain activity and more precisely power changes in certain frequency bands of the electroencephalogram (EEG) signal. Herein, these changes will be referred to as band power (BP) changes.

Due to their portability, high temporal resolution and relatively low cost, most of the currently used passive brain computer interface systems are EEG-based (Babiloni, 2019). The study from Babiloni (2019), for example, showed that BP features extracted from the EEG can successfully detect and distinguish the different mental states of the user, such as MWL and MF. They reported that an increase in MWL leads to a BP increase in the theta frequency band at frontal cortical areas with a simultaneous BP decrease in the alpha band at parietal areas (Babiloni, 2019). These results corroborate the findings in Holm et al. (2009); Stipacek et al. (2003), and Scerbo et al. (2003). Klimesch et al. (1999) reported that increasing MF is associated with a BP increase in the lower frequency bands (<12 Hz) and a BP decrease in the higher frequency bands (>12 Hz). Similar findings were observed in the study of Boksem et al. (2005). In another study, Käthner et al. (2014) found increasing alpha BP at parietal cortical areas and an increase in the theta BP at multiple widespread electrode positions, linked to elevated MF.

For mental state detection from EEG, most commonly used classifiers include linear discriminant analysis (LDA) and its variants (shrinkage or stepwise LDA), support vector machines and *k*-nearest neighbors (Liu et al., 2017; Acı et al., 2019; Lotfan et al., 2019; Lotte and Roy, 2019). Another classification approach is based on the so-called ensemble learning. The idea of this concept is to combine different classifiers such as LDA, support vector machines and artificial neural networks (Klosterman and Estepp, 2019) or use multiple base classifiers such as convolutional neural networks (Gao et al., 2019; Salimi et al., 2019; Zhang et al., 2019, 2021). In order to improve classification accuracy, spatial filtering techniques like the common spatial pattern (CSP) algorithm are used in combination with a linear classifier (e.g., LDA; Barachant et al., 2010). The CSP algorithm maximizes the class separability by calculating the eigenvalues of the covariance matrix of the classes (Ramoser et al., 2000). Another concept that makes use of the eigenvalues of covariance matrices is the Riemannian geometry (RG). As presented in Yger et al. (2017); Appriou et al. (2018), and Congedo et al. (2017), the RG can be employed for feature representation and learning, classifier design and calibration time reduction. Barachant et al. (2010, 2013b) showed that the RG can be also successfully used to classify motor imagery tasks. Furthermore, they showed that it can be easily expanded to the multi-class scenario and it can be combined with kernel-based machine learning methods due to the scalability of the Riemannian framework (Barachant and Bonnet, 2011; Barachant et al., 2013b).

In this work our aim was to detect high MWL and MF in participants performing cognitively demanding tasks. To mentally challenge and tire the participants, the letter *n*-back task (Kirchner, 1958) was used. For MWL and MF detection, we recorded brain activity for subsequent signal processing and data analysis. The brain signals were acquired non-invasively, using the EEG as the main measurement modality. Our main methodological approach relied on detecting changes on the covariance matrices of different EEG trials with respect to a reference state. Covariance matrices belong to the Riemannian manifold of the symmetric and positive definite matrices and the RG technique provides a framework to manipulate these matrices and measure their distances in their native space. Our signal analysis findings were supported by self-reporting and subjective ratings, as well as behavioral measures. Based on the literature of mental state monitoring systems, we explored the relationship between MWL, MF, and EEG BP changes. An increase in theta BP and a decrease in alpha BP is expected during high MWL, whereas an increase in alpha BP is expected during high MF. We hypothesized that these changes would also be projected on the Riemannian distances between resting and task-induced EEG signals in the alpha and theta band. This hypothesis was subsequently used as a basis for MWL and MF detection.

## Materials and Methods

### Participants

Twenty healthy subjects participated in this study (7 female and 13 male). The participants had normal or corrected to normal vision. Their age ranged from 21 to 31 years, with a mean of 26.15 years and a standard deviation of 2.6 years. The participants were informed about all the aspects of the experiment and voluntarily provided their written informed consent. All subjects were instructed to sit calmly on their chair and avoid (as much as possible) eye, head and body movements during the experiment.

### Experimental Design

**FIGURE 1:**
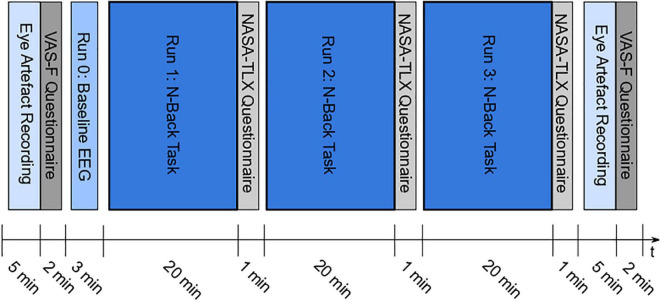
Experimental design – Paradigm for eye artifact recording at the beginning and at the end of the experiment, followed by the VAS-F questionnaire; Run 0: passive screening of the task to record the baseline EEG; Run 1, 2, and 3: performing letter *n*-back task, followed by NASA-TLX questionnaire.

The experimental design is illustrated in Figure 1. At the beginning and at the end of the experiment, a paradigm to intentionally record eye artifacts was presented, followed by the VAS-F questionnaire. In Run 0, the baseline EEG was recorded during a passive screening of the task (resting EEG). In run 1, 2, and 3, the participants had to perform the letter *n*-back task (Kirchner, 1958). After each task-run, the NASA-TLX questionnaire had to be filled in. The different parts of the experiment are described in more detail in the following sections. The paradigms for the eye artifact recording, the passive screening and the letter n-back task were developed and presented using MATLAB^®^ (Release 2019b, The MathWorks, Inc., Natick, MA, United States)^1^ and the Psychtoolbox (Psychophysics Toolbox Version 3)^2^. The participants were seated on a desk chair in front of a monitor. A Keyboard was used to perform the letter *n*-back task.

#### Eye Artifact Recording

Artifacts due to eye movements, blinks and saccades strongly affect the EEG signal, and should therefore be corrected (Keren et al., 2010; Plöchl et al., 2012). At the beginning and at the end of the experiment, a paradigm to intentionally record these eye artifacts was presented. The paradigm used for eye movement recording, as well as the employed eye artifact detection and correction algorithms were introduced by Kobler et al. (2017). The paradigm included four different conditions: rest, horizontal, vertical, and blink. *Rest:* A blue circle was presented on a black screen. The participants had to fixate the blue circle without moving or blinking. *Horizontal:* The blue circle was moving between the left and right side of the screen. The participants had to follow the blue circle with their eyes. *Vertical:* The participants had to follow the blue circle with their eyes moving up and down on the screen. *Blink:* The blue circle was shrinking and enlarging at a certain frequency. The participants had to blink according to that frequency.

#### Recording the Baseline Electroencephalogram

In Run 0, a passive screening of the task was conducted. The participants were instructed to calmly look at the screen where the paradigm was presented, but without performing the task. In this way, the resting EEG of the participants was recorded, which was used as baseline EEG in the signal processing and data analysis part. The BP changes for detecting increasing MWL and MF were calculated based on the differences between the baseline EEG and the task-run EEG.

#### Performing the *N*-Back Task

In run 1, 2, and 3, the participants had to perform the letter *n*-back task with varying difficulty. The paradigm of the task was designed to exhaust and tire the participants, in order to elicit an increase in MWL and MF.

The letter *n*-back task consisted of a sequence of 20 letters. The goal of the task was to identify target letters within the sequence. A target letter was defined as follows: if the currently presented letter was the same as *n* letters back, the current letter was assigned as the target letter. In each sequence, five target letters were included. In order to avoid creating short words, which would facilitate the task, only consonants were used. During the experiment, three different *n*-back tasks were presented: 1-back, 2-back, and 3-back.

**FIGURE 2:**
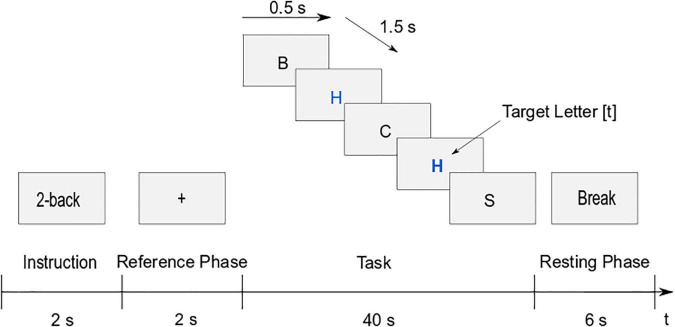
Trial structure of the letter *n*-back task (example 2-back): Instruction (2 s), reference phase (2 s), task (40 s), and resting phase (6 s).

The trial structure of the task runs can be seen in Figure 2. At the beginning of the trial, the instruction of the current task was presented for 2 s in order to inform the participants which *n*-back task they had to perform. In the reference phase, a fixation cross was shown for 2 s. The task itself had a duration of 40 s. Each letter was presented for 0.5 s, with 1.5 s pause between the letters. During the pause, the fixation cross was shown again to avoid random eye movements. If a target letter was identified, the participants had to press the *t* key. Once the task was completed, there was a 6 s break before the next trial. Each of the three conditions of the task-run (1-back, 2-back, and 3-back) was presented eight times, which led to 24 trials per run. The tasks were presented in a pseudo-randomized order, with the restriction of a maximum of two consecutive trials with the same task. One trial lasted 50 s, resulting in a total duration of 20 min per run.

#### Visual Analog Scale to Evaluate Fatigue Severity and NASA-TLX Questionnaires

The questionnaires were used to support the findings in the brain signals and evaluate whether the experiment was demanding enough to elicit high MWL and MF. The VAS-F questionnaire had to be filled in by the participants at the beginning and at the end of the experiment, after the eye artifact correction paradigm. This questionnaire evaluated the self-reporting and subjective rating of MF in the participants and consisted of 18 questions concerning individual levels of fatigue (e.g., feeling drowsy, tired, and worn out) and energy (e.g., feeling active, vigorous, and energetic) in ratings from 0 to 10. The NASA-TLX questionnaire had to be filled in by the participants after each task-run, in order to evaluate the self-reporting and subjective rating of MWL. This questionnaire consisted of six questions concerning workload (ratings from 0 to 20).

### Signal Acquisition

For the recording and amplification of the brain signals and the eye artifacts a mobile amplifier (LiveAmp; Brain Products GmbH, Gilching, Germany) was used. The amplifier was connected to the electrodes and placed in a pocket of the cap on the back of the participant’s head. The amplified signals were preprocessed (50 Hz notch filter) and sent via Bluetooth connection to a personal computer. As a recording software, the BrainVision Recorder (Brain Products GmbH, Gilching, Germany) was used. The EEG signals and the electroocculgram (EOG) were recorded by 32 active electrodes at a sampling rate of 500 Hz. The layout of the electrodes was modified from the Standard 32Ch actiCAP snap for LiveAmp (Easycap GmbH, Herrsching, Germany). To acquire the EEG signals, 28 electrodes were used at the following positions: Fp1, Fp2, F7, F3, Fz, F4, F8, FC5, FC1, FC2, FC6, T7, C3, Cz, C4, T8, CP5, CP1, CP2, CP6, P7, P3, Pz, P4, P8, O1, Oz, and O2. To record eye artifacts, three electrodes were used for the EOG. The electrodes were fixed with adhesive rings on the forehead (EOGM), on the left (EOGL), and on the right cheek (EOGR) of the participants. The ground electrode was placed at position Fpz, the reference electrode at position FCz. The electrode used for optional re-referencing was mounted at the right mastoid of the participants.

### Signal Processing

**FIGURE 3:**
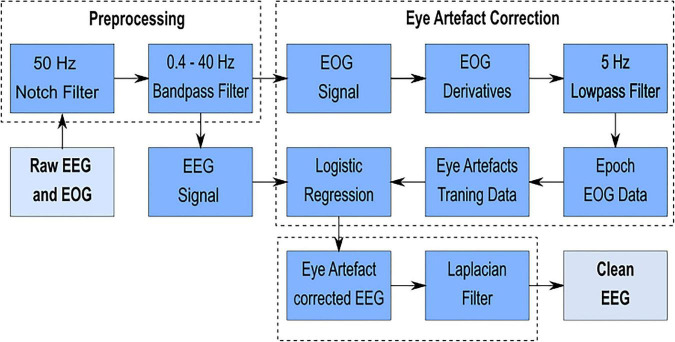
Signal processing chain – The individual steps from the raw EEG and EOG signal to the clean EEG signal can be divided into three blocks: preprocessing, eye artifact correction [adapted from Kobler et al. (2017)] and spatial resolution enhancement.

The individual steps of the signal processing chain are illustrated in Figure 3 and can be divided into three blocks: preprocessing, eye artifact correction [adapted from Kobler et al. (2017)] and spatial resolution enhancement. Signal processing was implemented in MATLAB^®^, supported by adapted functions from the EEGLAB toolbox^3^ (Delorme and Makeig, 2004). EEG and EOG and the paradigm markers generated with MATLAB^®^ were linked via lab streaming layer (LSL; Kothe, 2014) and recorded with the LabRecorder (default recording program for LSL)^4^. The recorded signals were saved to an extensible data format file.

#### Preprocessing

Fifty Hz power line interference was removed using a 50 Hz notch filter (Retdian and Shima, 2016). The EEG and EOG signals were additionally band-pass filtered between 0.4 and 40 Hz (zero-phase fourth-order Butterworth filter) in order to remove the unwanted direct current component (0 Hz), the low (<0.4 Hz) and the high (>40 Hz) frequency components.

#### Eye Artifact Correction

The eye artifact correction block was adapted from Kobler et al. (2017). The first step was to calculate the EOG derivatives from the three EOG electrodes (EOGL, EOGM, and EOGR): horizontal, vertical, and radial EOG derivatives. Next, a lowpass filter was applied on the EOG derivatives, in order to remove the unwanted higher frequency components. The applied filter was a zero-phase second order Butterworth filter with a cutoff frequency of 5 Hz. The lowpass-filtered EOG derivatives were divided into trials (epochs). The EOG paradigm markers were used to cut the continuous signal into epochs of the same length, as well as to distinguish between the four conditions (rest, horizontal, vertical, and blink). Noisy trials were excluded after visual inspection. With the information about the eye movements from the eye artifact paradigm and the EOG derivative epochs, a training data set was generated. The training data was fitted by the eye artifact correction algorithm and consisted of six different artifact classes: right, left, up, down, blink, and rest. The algorithm used a penalized logistic regression model to classify the eye artifacts and remove them from the EEG signal.

#### Spatial Resolution Enhancement

To further improve the spatial resolution, EEG was spatially filtered by applying an orthogonal derivation (Laplace filter; Hjorth, 1975).

### Data Analysis

After the preprocessing and artifact correction of the signals, the cleaned EEG signals were used for further data analysis. This section describes the steps that were followed to extract BP differences between task-run and baseline EEG in different frequency bands. We also present the methods that were employed to conduct statistical analysis and dataset classification, as well as the algorithms that were used to define and detect high MWL and MF.

#### Calculating Band Power Differences

To calculate BP, the EEG signals were first decomposed into theta (4–8 Hz) and alpha (8–13 Hz) frequency bands by applying a zero-phase fourth-order Butterworth bandpass filter. The continuous signals were then divided into single trials (epochs) and separated by the three task conditions (1-back, 2-back, and 3-back). BP was defined as the decadic logarithm of the power of the bandpass signal epoch during the task phase (40 s) of a single trial. BP calculation was performed for each trial of the task-run EEG in both theta and alpha frequency bands and for each condition (1-back, 2-back, and 3-back). In order to compute the BP changes after each single trial of the experiment, each BP result from the task-run EEG was subtracted by the mean of the baseline EEG (averaged over all three trials). The mean of the baseline EEG was used to compare the BP results from signals of equal length. For the grand average analysis, the participants were divided into two groups according to their task performance: Group 1 (high performers) and Group 2 (low performers). The segmentation was done by the median split of the task performance (and specifically the performance accuracy) of the participants. The median split is a very common form of dichotomization that has been extensively used in various studies to infer groups based on continuous variables.

#### Statistical Analyses

For the statistical analyses, the BP changes of each condition (1-back, 2-back, and 3-back) and each run (run 1, run 2, and run 3) were inspected. The BP changes of the individual trials were averaged over a whole run, for each condition separately. Furthermore, the individual channels were aggregated into four regions of interest (ROIs): *Frontal:* Fp1, Fp2, F7, F3, Fz, F4, and F8; *Central:* FC5, FC1, FC2, FC6, C3, Cz, and C4; *Parietal:* CP5, CP1, CP2, CP6, P7, P3, Pz, P4, and P8; *Occipital:* O1, Oz, and O2. Again, the participants were divided into two groups, according to their task performance. Statistical analysis was conducted for both alpha and theta frequency bands, separately. To investigate possible effects and interactions between the different conditions we ran a 3 × 3 × 4 mixed design repeated measures analysis of variance (ANOVA). The within factors included the runs (RUN: i.e., run 1, 2, and 3), the conditions (TASK: i.e., 1-back, 2-back, and 3-back) and the ROIs (ROI: i.e., frontal, central, parietal, and occipital). The between factors were generated from the two participant groups. To further investigate the influence of these parameters on the BP changes, a *post hoc* analysis was conducted. Within this analysis, the BP changes from each run, condition and ROI were compared, as well as the influence of the two participant groups (high and low performers). A bonferroni correction was used to control for multiple comparisons in the *post hoc* tests. Both the repeated measures of ANOVA and the *post hoc* analyses were performed with the free and open-source statistical software Jamovi^5^.

#### Detection of High Mental Workload and Mental Fatigue

For the detection of high MWL and MF, we applied a framework based on concepts of RG. RG has already been introduced to detect artifacts in EEG signals, by calculating the Riemannian distance of a signal to a defined threshold of the reference (baseline) signal (Barachant et al., 2013b). First, the covariance matrix of the reference signal (baseline EEG) was calculated as:

(1) $$C_{\text{ref}}=\frac{1}{N_{\text{ref}}-1}\cdot X_{\mathrm{re}\,f}\,X_{\mathrm{re}\,f}^{T}$$

The covariance matrix ($X_{\mathrm{re}\,f}\,X_{\mathrm{re}\,f}^{T}$) was scaled by the number of samples *N*_*ref*_ of the reference signal. Next, a window of 500 samples (i.e., 1 s) and a step size of 125 samples was applied to the reference signal. Note that the window and the step size was selected as an optimal tradeoff between temporal resolution and computational runtime. A small window would lead to biased covariance estimates. On the other hand, a longer window would provide lower temporal resolution and tracking performance. The step size defines the overlap between neighboring windows and affects both temporal resolution but also runtime. The smaller the step size the better the resolution but also the higher the computational workload. A step size of 125 was found to be appropriate to track changes in the alpha and theta band. For each window, the covariance matrix *C*_*win*_ was calculated similarly as in (1). The combined eigenvalues of the covariances *C*_*win*_ and *C*_*ref*_ were given as:

(2) $$\lambda =\text{eig}\,\left(C_{\text{win}}^{-1/2}\,C_{\mathrm{re}\,f}\,C_{\text{win}}^{-1/2}\right)$$

The Riemannian distance of the window to the reference signal (*D*_*R*_) was computed by summing up the logarithmic power of each eigenvalue λ_*n*_ and taking its square root (Förstner and Moonen, 2003):

(3) $$D_{R}=\sqrt{\sum_{n=1}^{N}{\text{log}}_{10}\,{\left(\lambda_{n}\right)}^{2}}$$

Equation (3) was tracked in time by estimating its value for each step throughout the whole reference signal. The same procedure was applied to the task-run EEG. The distance for each condition, in each run and for both frequency bands (i.e., theta and alpha) was calculated. Again, we used a window of 500 samples and a step size of 125 samples. For each step, the covariance matrix of the corresponding window and its eigenvalues were estimated using Eqs. (1) and (2). *C*_*ref*_ was defined as the covariance matrix of the reference signal (baseline EEG). The Riemannian distances of the eigenvalues (i.e., *D*_*T*_) were again computed based on Eq. (3).

To define an appropriate threshold for MWL and MF detection, Barachant et al. (2013a) used the following equation:

(4) $$\text{THRS}=\text{mean}\,\left(D_{R}\right)+2.5\cdot \text{std}\,\left(D_{R}\right)$$

Herein, however, we followed the standardization procedure suggested by Congedo (2013). The Riemannian distances are characterized by their asymmetric distribution. To make their distribution symmetric we used the standardized distances to the geometric mean with respect always to the reference signal. Specifically, for each run, we estimated the geometric mean (μ_ref_) and geometric standard deviation (σ_ref_) of the Riemannian distances of the reference signal,

(5) $$\mu_{\text{ref}}=e^{\left(\frac{1}{K}\,\sum_{k}\ln\left(D_{R}\right)\right)}$$

(6) $$\sigma_{\text{ref}}=e^{\left(\sqrt{\frac{1}{K}\,\sum_{k}{\left[\ln\left(\frac{D_{R}}{\mu_{\text{ref}}}\right)\right]}^{2}}\right)}$$

and used both Eqs. (5) and (6) to standardize the distances extracted from the task-run EEG as follows:

(7) $$z\,\left(D_{T}\right)=\frac{\ln\left(\frac{D_{T}}{\mu_{\text{ref}}}\right)}{\ln\left(\sigma_{\text{ref}}\right)}$$

Then, the standardized task-induced Riemannian distances (Eq. 7) were compared to a predefined threshold of:

(8) T⁢H⁢R⁢S=±2.5

To detect high MWL and MF, the Riemannian distances of each step through the task signal (task-run EEG) were averaged over trials and compared with the threshold of Eq. (8). If the average of the Riemannian distance reached or surpassed the threshold, high MWL and MF was detected in the participants. The distances were calculated in both frequency bands (theta and alpha).

## Results

### Grand Average Band Power Changes

**FIGURE 4:**
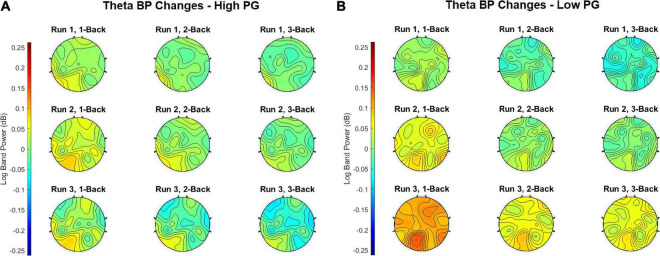
Grand average theta BP changes of the **(A)** high PG and **(B)** low PG. The task conditions were averaged over all trials at each run.

**FIGURE 5:**
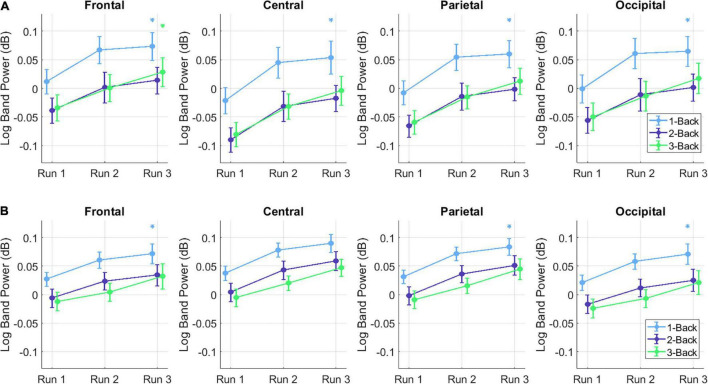
Mean ± SE of the **(A)** theta and **(B)** alpha BP changes in the low and high PG for each run, task (color-coded) and ROI (i.e., frontal, central, parietal, and occipital). Asterisks denote statistically significant differences (*p* < 0.05) between low and high PG. The color of the asterisk represents a specific task (i.e., 1-back: blue, 2-back: purple, or 3-back: green).

**FIGURE 6:**
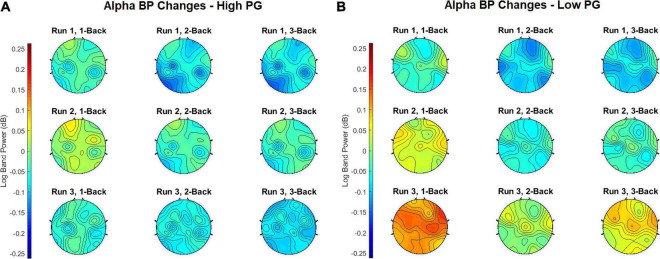
Grand average alpha BP changes of the **(A)** high PG and **(B)** low PG. The task conditions were averaged over all trials at each run.

In order to obtain the BP changes over time (i.e., runs), a grand average analysis (average over all participants) was conducted for the theta and the alpha frequency bands separately. The BP changes of each trial were averaged over each task condition (1-back, 2-back, and 3-back) at each run. Additionally, the BP changes were computed separately for the high and the low performance group (PG). The BP changes of the theta frequency band of the high performers are illustrated in Figure 4A, the BP changes of the low performers in Figure 4B. For both PGs, there was a slight BP increase in run 1 and run 2. For the high performers, however, the BP slightly decreased again in run 3, whereas the low performers exhibited a high BP increase. The most significant differences between high and low performers were found at the 1-back task in run 3 (Figure 5A). The BP changes for both PGs were most prominent at the parietal cortex. The grand average results of the BP changes in the alpha frequency band showed a slightly different behavior for both the high (Figure 6A) and the low performers (Figure 6B). For both PGs, there was a BP decrease in run 1, followed by a slight BP increase in run 2 (Figure 5B). As in the case of the theta frequency band, the BP of the high performers slightly decreased again in run 3, whereas the BP of the low performers showed a high increase. The most significant differences between high and low performers were found at the 1-back task in run 3 (Figure 5B). In contrast to the theta band, the BP changes for both PGs in the alpha band were most prominent at the central cortex. For both frequency bands, overall, the low PG showed a higher BP increase compared to the high PG.

#### Statistical Analyses

For the alpha band a significant main effect was found for TASK [*F*(1.68, 30.26) = 17.24, *p* < 0.001, ηp2 = 0.49], RUN [*F*(1.56, 28) = 22.16, *p* < 0.001, ηp2 = 0.55], and ROI [*F*(2.03,36.58) = 5.02, *p* = 0.011, ηp2 = 0.22]. The between subject factor PG did not reach significance [*F*(1,18) = 2.27, *p* = 0.15, ηp2 = 0.12].

For the *post hoc* tests, the different conditions were compared with each other using paired *t*-tests with Bonferroni correction. For the low performers, a statistically significant difference was detected only between run 1 and run 3 for both the theta [*t*(19) = −3.91, *p* < 0.01] and the alpha band [*t*(19) = −4.72, *p* < 0.01]. At the alpha band, the comparisons reached statistical significance at the 1-back task between run 1 and run 2 [*t*(19) = −4.13, *p* < 0.01] and between run 1 and run 3 [*t*(19) = −4.66, *p* < 0.01], as well as at the 3-back task between run 1 and run 3 [*t*(19) = −4.57, *p* < 0.01]. Statistically significant differences at the alpha band were found between run 1 and run 3 at the following ROIs: at the frontal [*t*(19) = −4.36, *p* < 0.01], the central [*t*(19) = −5.46, *p* < 0.01], the parietal [*t*(19) = –4.86, *p* < 0.01], and the occipital cortex [*t*(19) = −4.68, *p* < 0.01]. Additionally, the comparison between run 1 and run 2 showed statistical significance at the central cortex [*t*(19) = −4.19, *p* < 0.01]. For the theta-band a significant main effect was found for TASK [*F*(1.22, 22) = 17.24, *p* = 0.002, ηp2 = 0.39], RUN [*F*(1.79, 32.28) = 18.26, *p* < 0.001, ηp2 = 0.51], and ROI [*F*(1.60,28.74) = 9.49, *p* = 0.001, ηp2 = 0.35]. Furthermore a significant interaction effect was observed for RUN × ROI, [*F*(2.88, 51.28) = 3.49, *p* = 0.023, ηp2 = 0.16]. *Post hoc* analysis revealed significant differences in the frontal region between run 1 and run 2 [*t*(19) = 4.43, *p* = 0.021], in parietal region between run 1 and run 3 [*t*(19) = 5.10, *p* = 0.005] and in occipital region between run 1 and run 2 [*t*(19) = 4.67, *p* = 0.013] and run 1 and run 3 [*t*(19) = 5.01, *p* = 0.006]. The between subject factor PG did not reach significance [*F*(1,18) = 2.27, *p* = 0.15, ηp2 = 0.12].

### Subjective Ratings and Behavioral Measures

In addition to the BP changes of the EEG, subjective ratings and behavioral measures were also used to determine increasing MWL and MF. For the subjective ratings, VAS-F and NASA-TLX questionnaires were conducted before, during and after the experiments. As behavioral measures, the task performance accuracies and the response times were used.

#### Subjective Ratings

The VAS-F questionnaires were taken before and after the experiments and were divided into two categories: the fatigue level and the energy level. The highest possible score of both rating categories was scaled to 100% for comparison purposes. The grand average rating of the fatigue level increased from 24 to 54%, whereas the energy level decreased from 65 to 46% throughout the duration of the whole experiment. The NASA-TLX questionnaires were conducted after each run. Again, the highest possible rating score was scaled to 100%. The results of the individual subjects reveal a diverging behavior: For some, the task load increased during the experiment, for others the task load remained the same or even decreased. However, the grand average ratings of the task load increased continuously after each run (56, 63, and 68%, respectively).

#### Behavioral Measures

**FIGURE 7:**
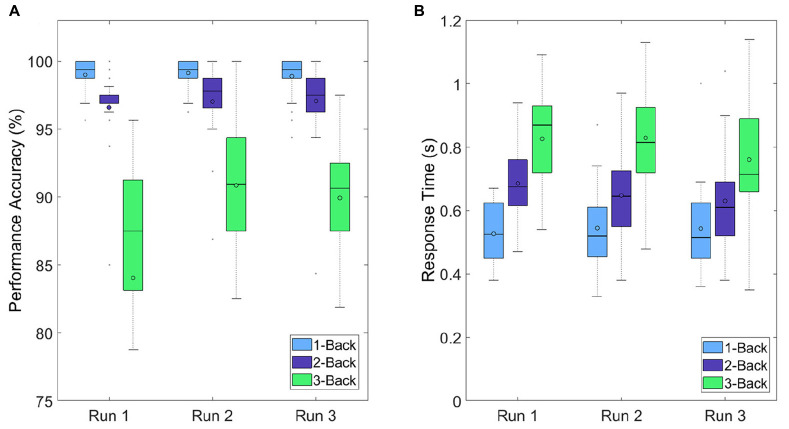
Boxplots depicting **(A)** performance accuracies and **(B)** response times over all participants for each run and each task condition.

The performance accuracies were calculated based on the number of correctly detected target and non-target letters during a task. An accuracy of 100% means that all five target letters were detected correctly (by pressing the *t* key) and none of the 15 non-target letters were identified as a target letter. The accuracies of each task condition were averaged over all trials at each run. The performance accuracies decreased with increasing task difficulty (i.e., increasing n), which means that the 1-back task yielded the highest performance accuracies (99, 99.13, and 98.88%) and the 3-back task the lowest (88.03, 90.84, and 89.94%). By comparing the performance accuracies between the runs, however, the results showed only small changes. For each task condition, there was a small increase in performance accuracy from run 1 to run 2 and a small decrease from run 2 to run 3 (Figure 7A). As a second performance measure, the response times of the subjects during the task execution were considered. Response time was defined as the time difference between the letter onset and the pressing of the *t* key. The response times of each task condition were averaged over all trials at each run. The response times increased with increasing task difficulty (increasing n), which means that the lowest and highest response times were detected during the 1-back task (0.53, 0.54, and 0.54 s) and the 3-back task (0.83, 0.83, and 0.76 s), respectively (Figure 7B). Similarly to the performance accuracies, only small changes occurred between the runs. During the 1-back task, there was a small increase from run 1 to run 3 (0.01 s). During the 2-back and 3-back task, however, we detected a small decrease from run 1 to run 3 (0.06 and 0.07 s).

### Mental Workload and Mental Fatigue Detection

**FIGURE 8:**
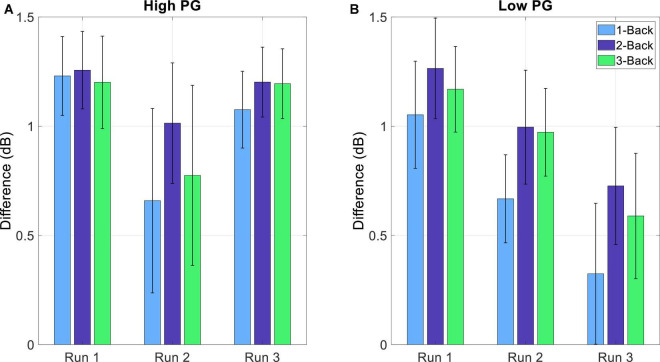
Average difference (and SE) between the threshold (THRS – Eq. 8) and the standardized Riemannian distances of the task-run EEG (Eq. 7) and for the **(A)** high and **(B)** low PG.

The detection of MWL and MF was implemented by using the RG. MWL and MF were defined as too high when the Riemannian distances of a task-run EEG reached or surpassed the threshold of Eq. (8). The differences between the threshold and the Riemannian distances are presented in Table 1. The distances of each window were averaged over all trials per task condition at each run. The Riemannian distances reached or surpassed the threshold (illustrated as 0 or negative difference, respectively) in eight subjects (gray highlighted rows). The number of high MWL and MF detections increased with increasing experiment duration (increasing run number). Four detections occurred at run 1, 7 at run 2, and 8 at run 3. The grand average differences of all subjects decreased with increasing run number (1.20, 0.84, and 0.88 for run 1, 2, and 3, respectively). Figure 8 shows the grand average differences for each PG separately. The differences between the threshold and the Riemannian distances of the low performers (1.16, 0.87, and 0.54 for run 1, 2, and 3, respectively) were lower than the differences of the high performers (1.22, 0.81, and 1.15 for run 1, 2, and 3, respectively). Furthermore, the differences of the low PG decreased continuously over time, whereas the differences of the high PG stayed the same within the experiment duration.

**TABLE 1:** Absolute differences between the threshold (Eq. 8) and the standardized Riemannian distances of the task-run EEG (Eq. 7) for the low and high PG.

Sub.	Difference between threshold and Riemannian distance (dB)									PG
Run	1			2			3			
Task	1	2	3	1	2	3	1	2	3	
EB1	1.78	1.88	1.28	1.14	1.36	0.89	1.17	1.17	0.99	Low
EI5	0.48	0.82	0.60	0.27	0.21	0.52	0.13	0.24	0.20	Low
EK2	0.97	0.98	1.09	0.81	1.10	0.97	0.74	0.97	0.94	High
EQ1	0.59	0.76	1.07	**−−0.13**	**−−0.21**	0.67	**–0**.**13**	0.08	0.23	Low
EQ9	1.84	1.70	1.50	1.66	1.67	1.46	1.47	1.49	1.22	Low
ES8	2.07	2.11	2.08	1.05	2.08	1.90	1.54	2.09	1.99	Low
EU6	2.00	1.97	1.74	1.84	1.68	1.43	1.61	1.91	1.70	High
EV2	1.43	1.93	2.02	**−−2.99**	**−−1.33**	**−−3.08**	0.93	0.86	1.37	High
EV4	1.59	1.57	1.54	1.60	1.55	1.68	1.59	1.66	1.72	High
EV6	**−−0.03**	**−−0.14**	0.02	0.14	0.50	0.28	**–0**.**09**	0.11	**–0**.**07**	Low
EW1	1.44	1.41	1.45	1.09	1.27	1.28	0.98	0.90	0.85	High
EW2	1.16	1.45	1.51	1.09	1.56	1.28	1.29	1.35	1.41	High
EW3	1.05	1.30	1.08	0.26	0.42	0.26	0.24	0.18	**–0**.**07**	Low
EW4	1.03	1.10	1.17	1.15	1.13	1.04	0.91	0.98	0.94	High
EW5	0.43	1.58	1.46	0.43	1.61	1.68	0.14	1.35	1.45	Low
EW6	0.47	0.43	0.62	**−−0.10**	0.26	0.28	0.40	0.62	0.63	High
EW7	1.50	0.42	**−−0.32**	1.23	1.58	1.43	1.33	1.56	1.42	High
EW8	1.94	1.93	1.91	1.87	1.82	1.84	2.05	2.01	1.96	High
EW9	1.26	1.37	1.43	1.19	1.32	1.09	**–1**.**55**	**–0**.**17**	**–0**.**64**	Low
EX2	**0.00**	0.63	0.48	**−−0.34**	0.53	0.37	**0**.**00**	0.40	0.20	High
AVG	1.15	1.26	1.19	0.66	1.01	0.86	0.74	0.99	0.92	
	1.20			0.84			0.88			

*Values in bold font-weight refer to instances where the Riemannian distance reached or surpassed the threshold.*

## Discussion

In order to detect increasing MWL and MF, the BP changes over time were analyzed at the individual task conditions (1-back, 2-back, and 3-back), ROIs (frontal, central, parietal, and occipital) and PGs (high and low performers). To corroborate the findings of the BP changes, the behavioral measures (performance accuracy and response time) and subjective ratings (VAS-F and NASA-TLX questionnaires) were inspected. Furthermore, the RG was applied to define the threshold for detecting high MWL and MF.

### Differences Between Performance Groups and Task Conditions

The results revealed differences in the BP changes between the low and the high PG for both frequency bands. While for the low performers a high BP increase was elicited over time, the high performers showed only small BP increases and even BP decreases between runs. Furthermore, the *post hoc* tests showed only a statistically significant difference at the low PG between run 1 and run 3. These findings led to the expected assumption that MWL and MF are correlated with task performance. Our results are in accordance with previous studies Käthner et al. (2014) and Roy et al. (2013) that show that task performance decreases with increasing MWL and MF.

Concerning the task-conditions, MWL and MF were expected to increase with increasing task difficulty. We also hypothesized that BP would increase with increasing *n* of the letter *n*-back task. The results in Figures 4–5, however, show that the highest BP increase at both frequency bands was elicited at the 1-back task. A possible explanation of this outcome could be that the 1-back task was very easy to perform in relation to the other tasks, and the participants lost concentration and focus on the task. This theory can be supported by the BP increase at multiple, widespread cortical sites, which indicates that more cortical areas were activated during the 1-back task.

### Influence of Mental Workload and Mental Fatigue at the Investigated Regions of Interests

The topographical plots in Figures 4, 5 show that the theta BP increased at the frontal cortex for both PGs as suggested in previous studies (Scerbo et al., 2003; Stipacek et al., 2003; Holm et al., 2009; Babiloni, 2019). The strongest theta BP increase, however, occurred at the parietal cortex, which is not in line with previous findings of MWL increase. This can be explained by the overlapping BP increase toward the end of the experiment, caused by increasing MF as suggested by Boksem et al. (2005) and Klimesch et al. (1999). Since the exact cortical region influenced by increasing MF is not specified in the literature, this outcome leads to the assumption that increasing MF induces a higher theta BP increase at the parietal cortex than at other cortical areas. The *post hoc* test comparisons revealed no statistical significance in the theta band. The lowest *p*-values for each ROI, however, were achieved again between run 1 and run 3.

The results of the BP changes at the alpha band can be observed in Figures 6, 5. The BP decrease at the parietal sites for both PGs at run 1 supports previous findings concerning MWL increase (Scerbo et al., 2003; Stipacek et al., 2003; Holm et al., 2009; Babiloni, 2019). However, an alpha BP decrease was also observed at frontal and central sites. At run 2, increased alpha BP was detected at various cortical areas, indicating possibly increased MF (Klimesch et al., 1999; Boksem et al., 2005). This theory was confirmed by observing the differences between the PGs at run 3: While the high performers exhibited only small increases and even decreases in their alpha BP, there were high alpha BP increases in the low performers. It appears that the lower task performance of the low PG was an aftereffect of increased MF, expressed as a higher alpha BP increase especially at the fronto-central cortical areas. In contrast to the theta band, the *post hoc* test comparisons revealed statistically significant differences between run 1 and run 3 for each ROI. These results suggest that the influence of increasing MWL and MF is higher in the alpha band than in the theta band, which can also be seen at the topographical plots of the BP changes.

### Subjective Ratings and Behavioral Measures

The outcome of the VAS-F and NASA-TLX questionnaires suggest that the participants experienced an increase in their subjective workload and fatigue level and a decrease in their energy level during the experiment. Although these results indicate the influence of a cognitive demanding task on the mental state of the participants, as suggested by Käthner et al. (2014) and Roy et al. (2013), the increases of the fatigue and workload level (and the decrease of the energy level) were expected to be higher. This can be confirmed by looking at the performance accuracies: The results at a certain task condition were very similar at all 3 runs. The small increases from run 1 to run 2 can be attributed to learning effects, especially at the 3-back task. From run 2 to run 3, a drop in the performance accuracy was expected, but did not occur. It must be mentioned that the high task performance accuracies occurred due to the fact that there were only five targets opposed to 15 non-targets during a trial sequence. This means that detecting no target at all still led to a performance accuracy of 75%. The same behavior was projected on the response times: The response times for each task condition remained the same during the experiment or even decreased, instead of increasing.

Interpretation of subjective ratings must be done with caution (especially with a scale from 0 to 20). However, with an average of over 20 participants, the results can be considered meaningful. Overall, the participants experienced an increase of their fatigue and workload level, but not as high as expected. Together with the results of the behavioral measures (low increase or no increase at all), we concluded that the experiment was not demanding enough to elicit high MWL and MF in all participants. An improvement for further studies would be to prolong the duration of the experiment (e.g., by adding another run). Since the 3-back task is already quite demanding, adding a higher *n*-back task to increase the difficulty of the experiment is not recommended.

### Mental Workload and Mental Fatigue Detection

Most mental state monitoring systems use various classification algorithms in order to detect high MWL or MF. The classification approach was considered in this study as well, in the form of an LDA classifier in combination with a CSP filter based on BP features. However, the results showed no significant differences between the *n*-back tasks. To this end, the detection of high MWL and MF was implemented by using the RG. If the Riemannian distance of a task-run EEG reached or surpassed the threshold defined by the baseline EEG (expressed by 0 or negative difference values, respectively), the level of MWL and MF was defined as too high. Detection was conducted at the alpha band, since the influences of MWL and MF on the BP changes were revealed to be higher in the alpha than the theta band. Even though the detection rate was only 40%, this outcome is quite promising. The results confirm the assumptions that the experiment was not demanding enough for most of the participants. But still, we could observe a correlation between task performance and MWL/MF level.

Most of the detections occurred at run 3, indicating that fatigue and workload levels increased over time. This trend becomes even clearer when looking at the results of Figure 8. The differences between the Riemannian distance of the task-run and the threshold were averaged over each run and divided into the two PGs. For the high PG, there was a small decrease from run 1 to run 2, whereas from run 2 to run 3 the difference remained the same. In contrast, the differences of the low PG exhibited a continuous decrease during the experiment. This trend suggests that the low PG would have reached their MWL and MF limit if the experiment duration was longer.

Note that the Riemannian distances were averaged over all trials per task condition. In order to apply the RG approach on an online detection system for mental state monitoring, a single trial detection of MWL and MF would be desired. A potential error source of the RG approach is the influence of artifacts since the distance of an artifact influenced EEG signal may also surpass the threshold of the baseline EEG. To avoid these false positive detections, a comprehensive artifact correction pipeline must be implemented for online detection.

Channel selection and dimensionality reduction is an important aspect that we plan to include in future studies. By selecting topologically relevant electrodes and defining individualized alpha and theta frequency bands in each subject, the detection sensitivity of our proposed framework may improve further.

## Conclusion

The aim of this study was to detect high MWL and MF in participants performing a cognitive demanding task in the form of the letter *n*-back task. For the detection, RG was applied on BP features of the EEG. The results of the BP changes over time partly agreed with the findings in the presented literature. For increasing MWL, the theta BP continuously increased as expected at the frontal cortex but showed even higher increases at the parietal cortex. The alpha BP initially decreased according to the literature at the parietal cortex, but also at the frontal and central areas. At run 2, BP started to increase at multiple, widespread cortical areas. The theta and alpha BP increase toward the end of the experiment indicated increasing MF. It appears that the initial influence of increasing MWL on the BP changes is overlapped by the influence of increasing MF. Although this behavior was expected, a clear distinction between MWL and MF is not possible using only BP features.

The results of the subjective ratings and the behavioral measures revealed another limitation of this work: The experiment was not cognitively demanding enough to elicit high MWL and MF in all participants. This outcome was confirmed by the high BP differences between the low and high PG. A suggested improvement for further studies is to prolong the duration of the experiment, for example, by adding an additional run. The detection of high MWL and MF by using the Riemannian distances of the task run EEG showed promising results. High MWL and MF was detected mainly in the low PG. These findings are consistent with the observed BP changes at both the theta and the alpha frequency band, where increasing MWL and MF was only elicited for the low PG. Additionally, the averaged differences between the Riemannian distance of the task-run and the threshold of the low PG exhibited a negative correlation with experiment duration.

Overall, MWL and MF detection with the RG approach shows promising results. To validate the capabilities of our proposed detection algorithm, future studies must be conducted with a longer experiment duration and a larger number of participants. Future improvements regarding the methodological aspects of this work revolve around channel selection and individualized alpha/theta band definition for RD estimation. Further steps toward an online detection system for mental state monitoring require the implementation of a trial-wise detection algorithm and the application of an online artifact correction procedure.

## Data Availability Statement

The raw data supporting the conclusions of this article will be made available by the authors, without undue reservation.

## Ethics Statement

The studies involving human participants were reviewed and approved by the Medical University of Graz. The participants provided their written informed consent to participate in this study.

## Author Contributions

All the authors had full access to all the data in the study and took responsibility for the integrity of the data and the accuracy of the data analysis. SW and PR: conceptualization, methodology, and writing – original draft. PR: investigation and preprocessing and analysis. KK, SW, and GM-P: writing – review and editing. SW: supervision. All authors contributed to the article and approved the submitted version.

## Conflict of Interest

The authors declare that the research was conducted in the absence of any commercial or financial relationships that could be construed as a potential conflict of interest.

## Publisher’s Note

All claims expressed in this article are solely those of the authors and do not necessarily represent those of their affiliated organizations, or those of the publisher, the editors and the reviewers. Any product that may be evaluated in this article, or claim that may be made by its manufacturer, is not guaranteed or endorsed by the publisher.

## Footnotes

1
https://github.com/sccn/labstreaminglayer/wiki/

2
http://psychtoolbox.org/

3
http://www.sccn.ucsd.edu/eeglab/

4
https://github.com/sccn/lslarchived/wiki/LabRecorder.wiki/

5
https://www.jamovi.org/

## Abbreviations

ANOVA: Analysis of variance
BP: Band power
EEG: Electroencephalogram
EOG: Electrooculogram
LDA: Linear discriminant analysis
MWL: Mental workload
MF: Mental Fatigue
NASA-TLX: NASA Task load index
pBCI: Passive brain computer interface
PG: Performance group
ROI: Region of interest
RD: Riemannian distance
RG: Riemannian geometry
SE: Standard error
VAS-F: Visual Analog Scale to Evaluate Fatigue Severity.
