# Corrigendum: Mental State Detection Using Riemannian Geometry on Electroencephalogram Brain Signals

**DOI:** 10.3389/fnhum.2022.861120

**Published:** 2022-02-15

**Authors:** Selina C. Wriessnegger, Philipp Raggam, Kyriaki Kostoglou, Gernot R. Müller-Putz

**Affiliations:** ^1^Institute of Neural Engineering, Graz University of Technology, Graz, Austria; ^2^BioTechMed-Graz, Graz, Austria; ^3^Research Group Neuroinformatics, Faculty of Computer Science, University of Vienna, Vienna, Austria; ^4^Department of Neurology and Stroke, Hertie Institute for Clinical Brain Research, University of Tübingen, Tübingen, Germany

**Keywords:** EEG, mental workload, mental fatigue, band power features, Riemannian geometry

In the original article, there was a mistake in Figure 7 as published. Runs and tasks were mistakenly swapped. The corrected Figure 7 appears below.

**Figure 7:**
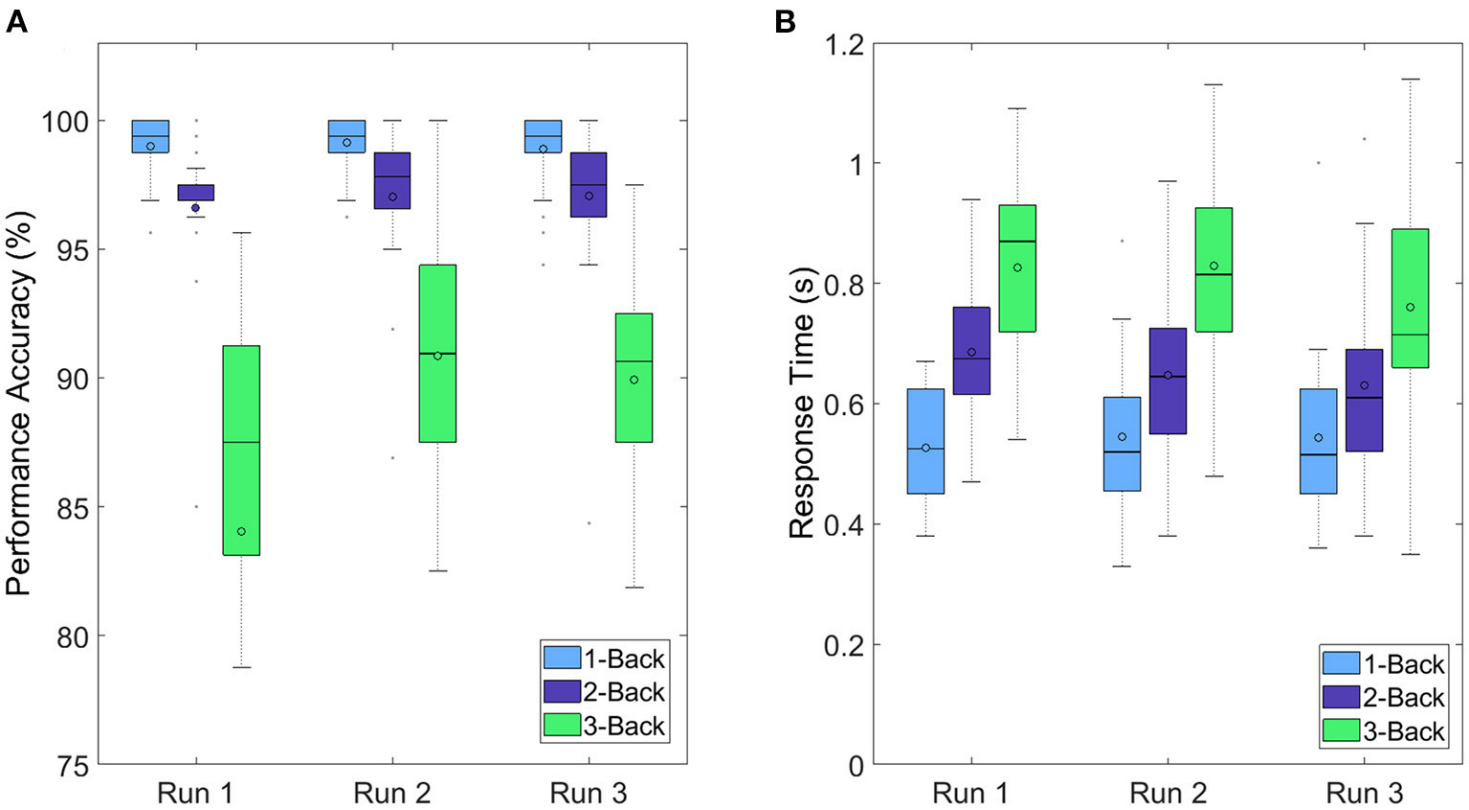
Boxplots depicting **(A)** performance accuracies and **(B)** response times over all participants for each run and each task condition.

The authors apologize for this error and state that this does not change the scientific conclusions of the article in any way. The original article has been updated.

## Publisher's Note

All claims expressed in this article are solely those of the authors and do not necessarily represent those of their affiliated organizations, or those of the publisher, the editors and the reviewers. Any product that may be evaluated in this article, or claim that may be made by its manufacturer, is not guaranteed or endorsed by the publisher.

